# Automatic grading of knee osteoarthritis with a plain radiograph radiomics model: combining anteroposterior and lateral images

**DOI:** 10.1186/s13244-024-01719-3

**Published:** 2024-06-13

**Authors:** Wei Li, Jin Liu, Zhongli Xiao, Dantian Zhu, Jianwei Liao, Wenjun Yu, Jiaxin Feng, Baoxin Qian, Yijie Fang, Shaolin Li

**Affiliations:** 1 Department of Radiology, The Fifth Affiliated Hospital of Sun Yat-sen University, Zhuhai, Guangdong Province China; 2 Huiying Medical Technology (Beijing), Huiying Medical Technology Co., Ltd., Room A206, B2, Dongsheng Science and Technology Park, Haidian District, Beijing, 100192 China

**Keywords:** Knee osteoarthritis, Radiomics, Grading, Radiograph, X-ray

## Abstract

**Objectives:**

To establish a radiomics-based automatic grading model for knee osteoarthritis (OA) and evaluate the influence of different body positions on the model’s effectiveness.

**Materials and methods:**

Plain radiographs of a total of 473 pairs of knee joints from 473 patients (May 2020 to July 2021) were retrospectively analyzed. Each knee joint included anteroposterior (AP) and lateral (LAT) images which were randomly assigned to the training cohort and the testing cohort at a ratio of 7:3. First, an assessment of knee OA severity was done by two independent radiologists with Kallgren–Lawrence grading scale. Then, another two radiologists independently delineated the region of interest for radiomic feature extraction and selection. The radiomic classification features were dimensionally reduced and a machine model was conducted using logistic regression (LR). Finally, the classification efficiency of the model was evaluated using receiver operating characteristic curves and the area under the curve (AUC).

**Results:**

The AUC (macro/micro) of the model using a combination of AP and LAT (AP&LAT) images were 0.772/0.778, 0.818/0.799, and 0.864/0.879, respectively. The radiomic features from the combined images achieved better classification performance than the individual position image (*p* < 0.05). The overall accuracy of the radiomic model with AP&LAT images was 0.727 compared to 0.712 and 0.417 for radiologists with 4 years and 2 years of musculoskeletal diagnostic experience.

**Conclusions:**

A radiomic model constructed by combining the AP&LAT images of the knee joint can better grade knee OA and assist clinicians in accurate diagnosis and treatment.

**Critical relevance statement:**

A radiomic model based on plain radiographs accurately grades knee OA severity. By utilizing the LR classifier and combining AP&LAT images, it improves accuracy and consistency in grading, aiding clinical decision-making, and treatment planning.

**Key Points:**

- Radiomic model performed more accurately in K/L grading of knee OA than junior radiologists.
- Radiomic features from the combined images achieved better classification performance than the individual position image.
- A radiomic model can improve the grading of knee OA and assist in diagnosis and treatment.

**Graphical Abstract:**

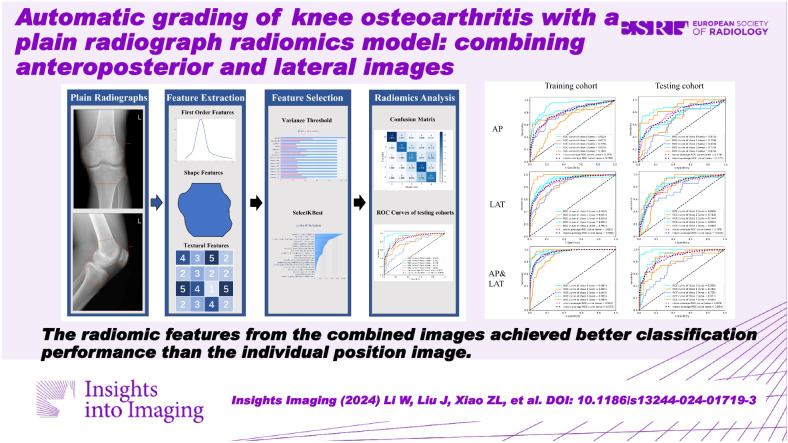

## Background

Knee osteoarthritis (OA) is a prevalent degenerative joint disease that impacts a large number of individuals globally [1]. Its main features include knee hyperosteogeny, degeneration, and destruction of articular cartilage [2]. In the early stage, knee OA patients may not experience obvious symptoms. However, as the disease progresses, they may experience knee joint pain, tenderness, limited joint mobility, and joint swelling [3]. In China, approximately 120 million individuals are affected by knee OA. The overall prevalence of primary knee OA among individuals aged over 40 is 17%, while among those aged over 75, it reaches a staggering 80% [4]. This prevalence is gradually increasing due to the aging of the population. Without proper diagnosis and treatment, knee OA can have a detrimental impact on individuals’ quality of life and work efficiency. Additionally, it places a substantial burden on families and society as a whole.

Radiographs are a commonly utilized method for evaluating the severity of knee OA, primarily due to their widespread availability and relatively low cost [5]. The grading of knee OA severity plays a crucial role in clinical decision-making and treatment planning. However, traditional radiographic grading systems, such as the Kellgren–Lawrence (K/L) grading system [6], suffer from subjectivity and poor inter-observer agreement. This is primarily due to limitations and inconsistencies in examination methods and technical levels, which directly impact disease judgment and result in inconsistency in treatment plans [5]. Therefore, there is an urgent need to develop quantitative evaluation methods for knee joint images and accurately grade knee OA.

In recent years, radiomics has emerged as a promising approach in the field of medical imaging for grading and classifying knee OA [7]. Radiomics is a field that involves extracting quantitative features from medical images to capture subtle changes in joint space and bone density. It enables a more detailed analysis and characterization of the underlying pathology associated with knee OA [8–10]. These features can be used to develop grading models for knee OA severity, providing a more objective and reproducible method for assessment. While radiomic methods have been extensively applied in tumor identification and prognosis prediction for various body systems, their application in knee OA has been relatively limited [11–15]. In a previous study [16], we successfully used a radiomic model to classify knee OA into OA and non-OA categories, achieving a high classification efficiency.

In this study, we constructed a radiomic model using anteroposterior (AP) and lateral (LAT) knee plain radiographs. Our aim was to evaluate the effectiveness of combining information from both AP and LAT (AP&LAT) images in grading knee OA, building upon previous research.

## Materials and methods

The research received ethical approval from the Institutional Ethics Committee at the Fifth Affiliated Hospital of Sun Yat-sen University and adhered to the principles outlined in the Declaration of Helsinki. As it is a retrospective study, the requirement for informed consent was waived by the ethics committee.

### Study population

**Fig. 1:**
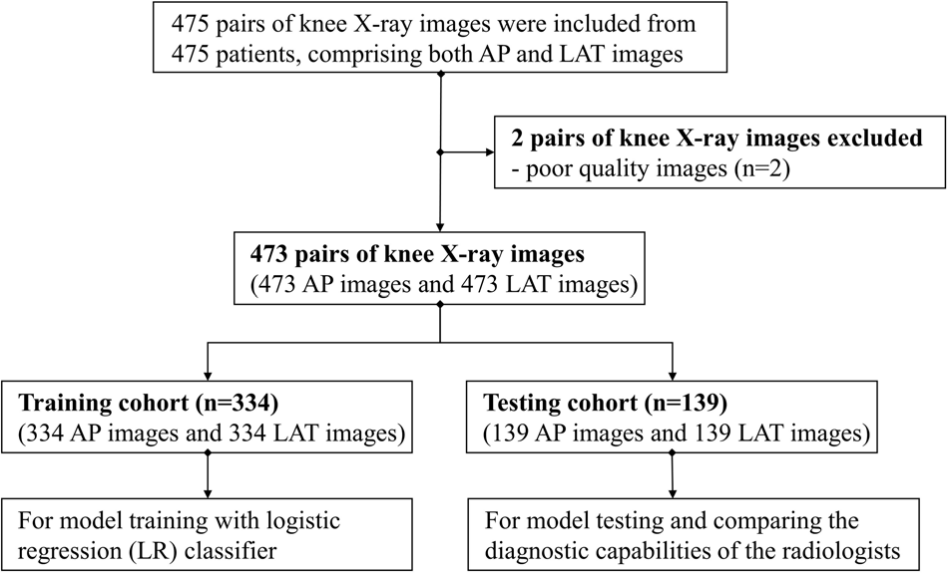
The workflow of patient enrollment and distribution

The study was conducted at the Fifth Affiliated Hospital of Sun Yat-sen University in Zhuhai, China, and specifically focused on adult patients with closed epiphyses who underwent knee X-ray imaging between May 2020 and July 2021. For inclusion, individuals above 18 years old who underwent AP&LAT knee X-ray imaging with a supine position in a single examination were considered. Patients who fulfilled any of the exclusion criteria outlined below were excluded from the study: (1) presence of tumors in the knee joint; (2) knee fractures; (3) congenital deformities of the knee joint; (4) other types of inflammatory arthritis such as gouty arthritis and rheumatoid arthritis; (5) poor-quality X-ray images; and (6) prior knee surgery. The workflow of patient enrollment and distribution is in Fig. 1.

### X-ray image acquisition and radiological evaluation

**Fig. 2:**
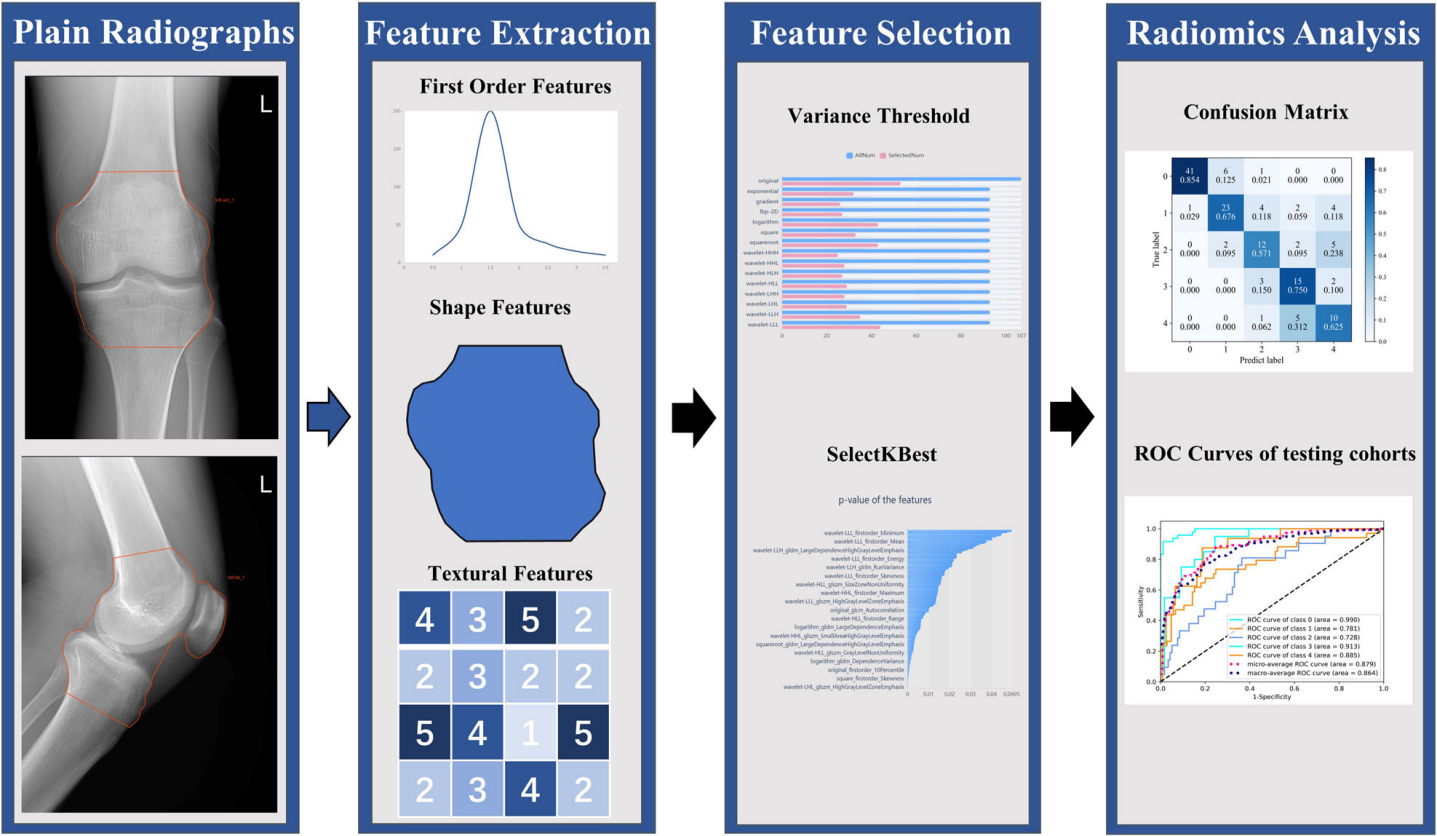
Regions of interest (ROIs) were manually delineated on AP&LAT plain radiographs. From these X-ray images, radiomic features were then extracted by quantifying their intensity, shape, and texture. The feature selection process was conducted in two stages. Subsequently, logistic regression (LR) models were employed to assess the grading performance of the selected radiomic features. The accuracy of the model was evaluated using receiver operating characteristic (ROC) curves

The image processing pipeline of this study is presented in Fig. 2. The X-ray images in their original form were in the DICOM format, obtained from a variety of imaging devices with differing dimensions. The study utilized desensitization throughout the process. *Z*-scores were used to standardize the images into a normal distribution while values outside of the 1% and 99% limits were trimmed to remove differences in index dimensions. The grading assessment was carried out by two musculoskeletal radiologists, W.L. and Y.F., who, respectively, possess 5 and 9 years of diagnostic experience. The K/L grading, which employs a numerical scale of 0 to 4 to denotate the magnitude from normal to very severe knee OA, was used as the reference standard [6]. The two radiologists graded all patients’ images separately according to the K/L grading criteria. To ensure objectivity, the radiologists were provided with no demographic or clinical information during the assessment. Finally, any inconsistencies that arose during the classification, were resolved by way of discussions aimed at finding consensus.

### Manual segmentation and feature extraction

**Fig. 3:**
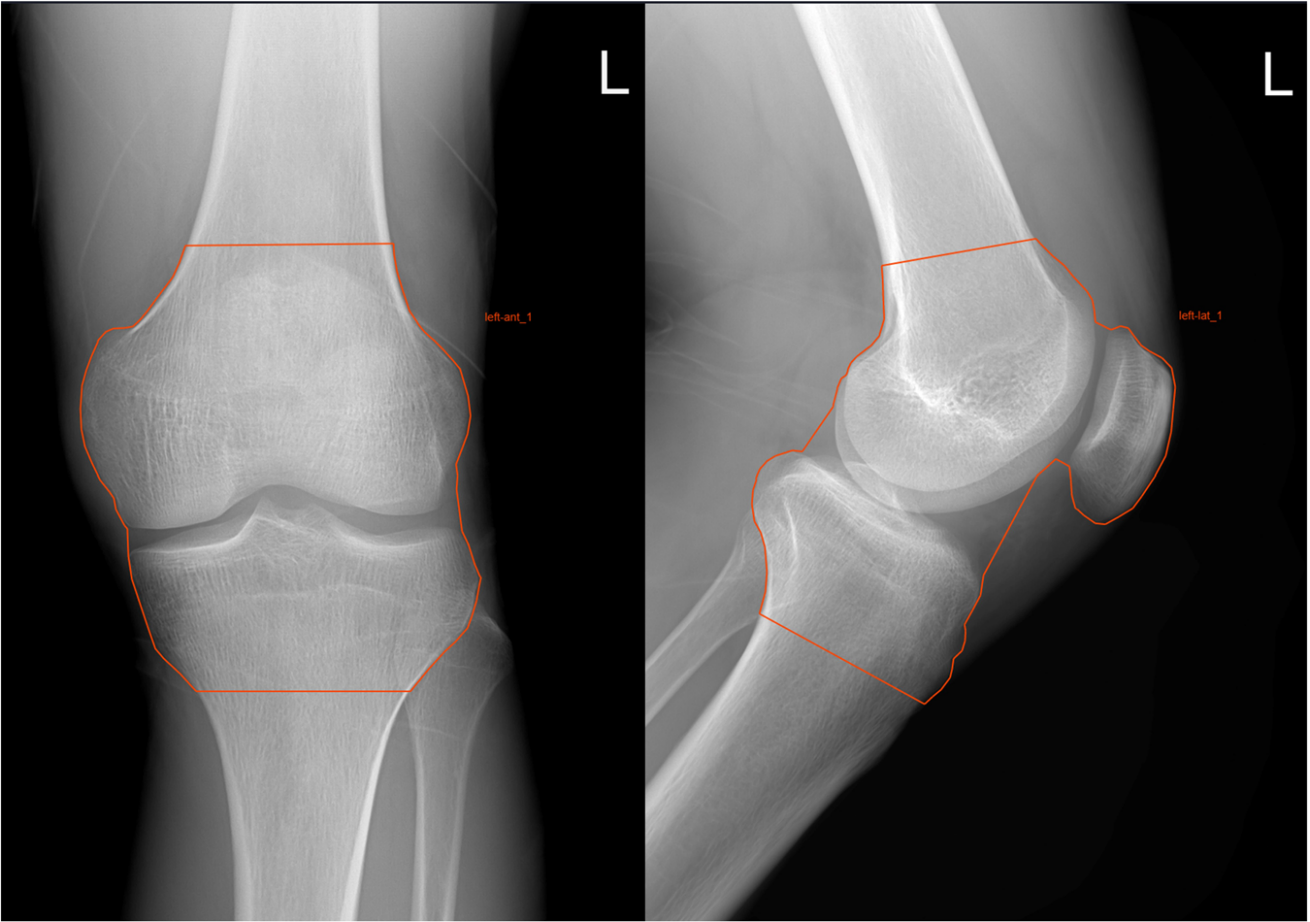
Schematic illustration of the manual ROI segmentation process

The region of interests (ROIs) encompassing the patella, femur, medial and lateral tibial condyles, and corresponding joint space (Fig. 3) were manually delineated by two experienced radiologists, reader 1 (J.F.) and reader 2 (J.L.), with 3 and 6 years of diagnostic experience, respectively. When delineating the ROI, it was specified that the distance from the articular surface to the lower femur was 6 cm (must include the patella) and the distance from the articular surface to the upper tibia was 3 cm. The joint space must include the medial tibiofemoral joint, the lateral tibiofemoral joint, and the patellofemoral joint. The Radcloud software (v.7.8, developed by Huiying Medical Technology Co., Ltd, China) was utilized to delineate the ROIs and extract/select radiomic features. The identified characteristics were classified into four groups. The first group, known as first-order features, describes the fundamental geometric attributes of lesions, including their size, shape, and surface roughness. The second group, shape-based features, illustrates the geometric properties of the lesions. The third group, texture features, defines the spatial distribution of the ROI pixels and highlights the spatial heterogeneity, including grayscale granularity, variations, and image roughness. Lastly, the fourth group, wavelet transform-based features, provides multi-resolution image description information obtained through wavelet transformation of the original image.

### Radiomic feature selection and dimensionality reduction

To assess the repeatability of ROI delineation, a random sample of 50 cases was selected from the collected data. Inter-observer agreement was evaluated by two radiologists, referred to as reader 1 (J.F.) and reader 2 (J.L.), who independently segmented the ROIs without knowledge of each other’s results. In order to assess the intra-observer agreement, reader 1 performed segmentation on the same set of 50 cases previously used. The obtained eigenvalues were then compared to the data collected a month ago. The reliability was evaluated using the intraclass correlation coefficient (ICC). These features were chosen based on evaluations from both intra-observer and inter-observer assessments. Only features that demonstrated excellent stability, with ICC values exceeding 0.75, were selected for subsequent dimensionality reduction analysis. The dimensionality reduction process consisted of two stages. The first stage involved selecting features with a variance value higher than 0.8. In the final analysis, features with a *p*-value below 0.05 were chosen using the SelectKBest method.

### Development and evaluation of models

The data was randomly split into training and testing cohorts using a ratio of 7:3. In the training cohort, 70% of the data was used to train the model, while the remaining 30% was reserved for independent testing. Logistic regression (LR) classifiers were used to train radiomic models, and their performance was evaluated based on the results from the testing cohort. The radiomic model was evaluated using a set of performance metrics, including accuracy (ACC), precision, recall, F1 score, and receiver operating characteristic (ROC) curve analysis, in both the training and testing cohorts. The discrimination performance of the final established models was assessed by quantifying both the ROC curve and the area under the curve (AUC) value.

Finally, we compared the diagnostic capabilities of the radiologists and the best-performing model by evaluating the accuracy in the testing cohort. We recruited two radiologists, D.Z. and Z.X., who have 4 and 2 years of musculoskeletal diagnostic experience, respectively. They were not involved in annotating the training cohort.

### Statistical analyses

Statistical analyses were conducted using SPSS 25.0 (IBM, Armonk, NY, USA) and R software (version 4.1.2, R Foundation; Vienna, Austria). Categorical variables were compared using either Pearson’s chi-square test or Fisher’s exact test. Continuous variables were compared using the *t*-test or Mann–Whitney *U*-test, as appropriate. The normality of continuous data was assessed using the Kolmogorov–Smirnov test. Data that followed a normal distribution were analyzed using the *t*-test and reported as mean ± standard deviation. Non-normal data were analyzed using the Mann–Whitney *U*-test, and skewed distribution data were presented as median (upper and lower quartiles). The AUCs of different models were compared using the DeLong test, and two-tailed *z*-tests were used to compute *p*-values. A significance level of 0.05 was considered statistically significant. Abbreviations were defined upon their first use, and standard formatting was maintained throughout the document.

## Results

### Demographic characteristics of patients

A total of 475 pairs of knee X-ray images were included from 475 patients, comprising both AP&LAT images. Two participants were excluded from the analysis due to poor-quality images. The remaining 473 patients (177 men and 296 women) were split into two cohorts: 334 in the training cohort and 139 in the testing cohort. No significant differences in age or gender were observed between the two cohorts (*p* = 0.481 and *p* = 0.349, respectively). A summary of the patients’ demographic characteristics is presented in Table 1.

**Table 1:** Baseline characteristics of the study population

Characteristic	Training cohort (*N* = 334)	Testing cohort (*N* = 139)	*p*-value
Age (years)^a^	54.25 (27.58)	49.50 (31.82)	0.481
Sex^b^			0.235
Male	122 (36.5)	55 (39.6)	
Female	212 (63.6)	84 60.4)	
Grade^b^			
0	114 (34.1)	48 (34.5)	
1	81 (24.2)	34 (24.5)	
2	51 (15.3)	21 (15.1)	
3	48 (14.4)	20 (14.4)	
4	40 (12.0)	16 (11.5)	

^a^Data is means, with SDs in parentheses

^b^Data is number of patients, with percentages in parentheses

### Segmentation and feature selection

**Fig. 4:**
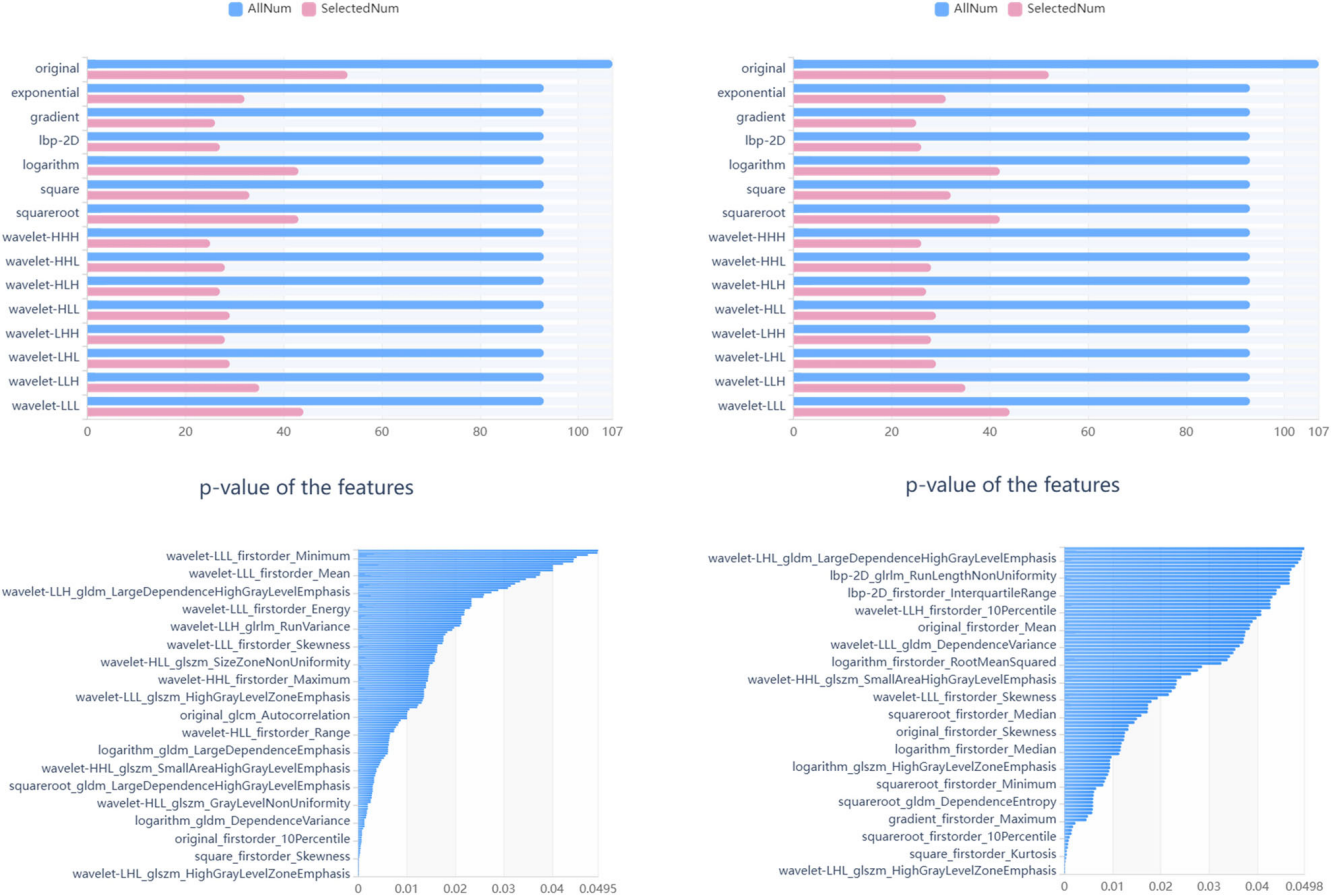
The process of reducing the dimensionality of AP (left) and LAT (right) images by selecting the variance threshold and using SelectKBest

An objective assessment of two radiologists’ ROI delineation (J.F. and J.L.) yielded an ICC value of 0.91. A total of 1409 radiomic features were selected for subsequent dimensionality reduction analysis. In the AP images, 496 radiomic features were originally extracted from the training cohort and 94 were further selected through SelectKBest following variance thresholding. Similarly, for the LAT images, a variance threshold technique was utilized to extract 502 radiomic features, out of which 148 features were selected through SelectKBest (Fig. 4).

### Performance of radiomic models

**Fig. 5:**
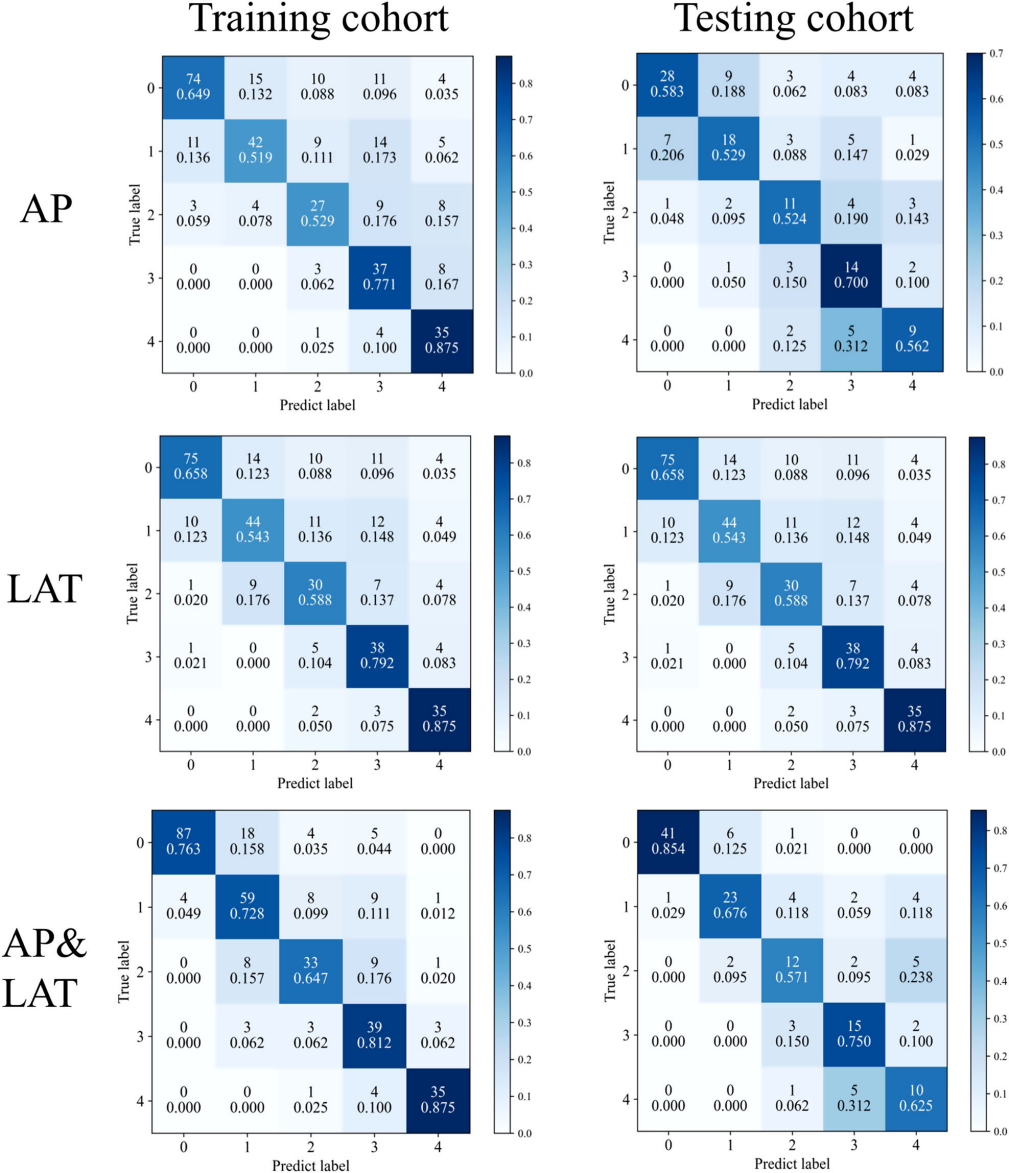
Confusion matrices for the network models compared on the training and testing cohorts are displayed on the left and right sides, respectively. The AP model, LAT model, and AP&LAT combined model results are presented in top-to-bottom order. The figures in the confusion matrices represent the percentage of the predicted class

**Fig. 6:**
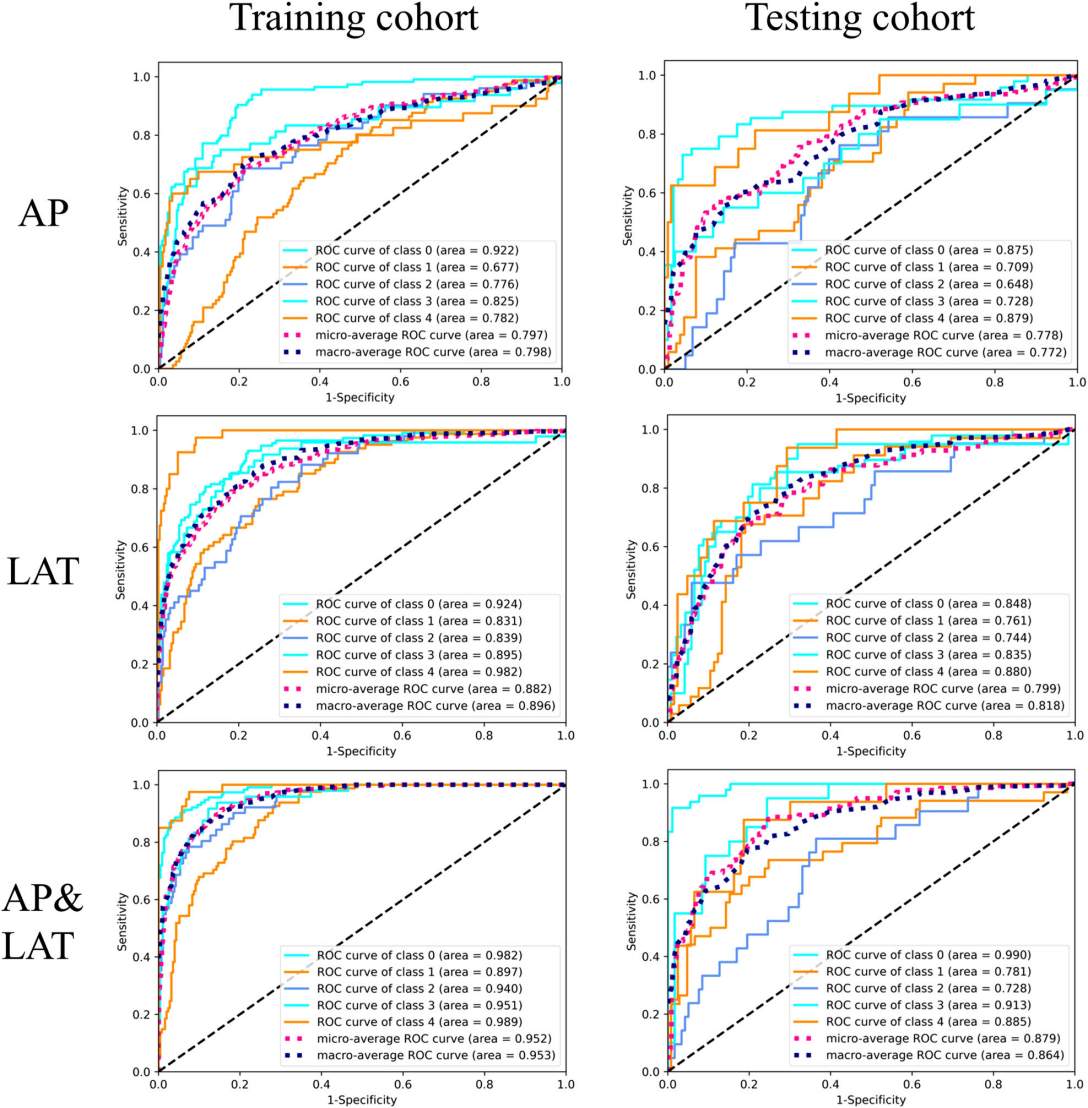
Five-class ROC curves for the training cohort (left) and testing cohort (right) are shown in three different models. Class 0 represents the normal group, Class 1 denotes the mild group, Class 2 indicates the moderate group, Class 3 signifies the severe group, and Class 4 represents the very severe group. The ROC curves of micro-average and macro-average are shown as dashed lines, indicating the overall discriminability of the five-class classification based on the AP model, the LAT model, and the combined AP&LAT model

Tables 2 and 3 present the diagnostic proficiency of three distinct models for the training and testing cohorts. Amongst them, the AP&LAT model outperformed the others. The AUC (macro/micro) values for the training cohort in AP, LAT, and AP&LAT models were 0.798/0.797, 0.896/0.882, and 0.953/0.952, respectively. For the testing cohort, the overall accuracy of AP, LAT, and AP&LAT models were 0.576, 0.626, and 0.727, respectively. The macro and micro precision, recall, and F1 scores for the AP&LAT model in the testing cohort were 0.678/0.750, 0.69 /0.727, and 0.683/0.734, respectively. The confusion matrices for the three models applied on the training and testing cohorts indicate that the AP&LAT model obtained the highest percentage of predictions (Fig. 5). The AUC value (macro/micro) of 0.864/0.879 for the AP&LAT model was found to be statistically significant, indicating a difference from the other two models in the testing group. The Delong test showed that the AP&LAT model outperformed both the AP model, with an AUC value of 0.772/0.778, and the LAT model, with an AUC value of 0.818/0.799, at *p* < 0.05. The ROC curves for the five-class testing cohort were analyzed in three different models and showed that the AP&LAT combined model had the best classification performance with AUCs of 0.990, 0.781, 0.728, 0.913 and 0.885 from class 0 to 4 (Fig. 6).

**Table 2:** Overall performance of different models based on training cohort

Model	Precision (macro/micro)	Recall (macro/micro)	F1 score (macro/micro)	AUC (macro/micro)	Accuracy	*p-*value
AP	0.629/0.677	0.669/0.644	0.632/0.645	0.798/0.797	0.644	< 0.05
LAT	0.652/0.692	0.691/0.665	0.660/0.667	0.896/0.882	0.665	< 0.05
AP&LAT	0.753/0.781	0.765/0.757	0.753/0.763	0.953/0.952	0.757	Reference

**Table 3:** Overall performance of different models based on testing cohort

	AP			LAT			AP&LAT		
	Precision	Recall	F1 score	Precision	Recall	F1 score	Precision	Recall	F1 score
Class 0	0.778	0.583	0.667	0.868	0.688	0.767	0.976	0.854	0.911
Class 1	0.600	0.529	0.562	0.543	0.559	0.551	0.742	0.676	0.708
Class 2	0.500	0.524	0.512	0.545	0.571	0.558	0.571	0.571	0.571
Class 3	0.438	0.700	0.538	0.500	0.700	0.583	0.625	0.75	0.682
Class 4	0.474	0.562	0.514	0.562	0.562	0.562	0.476	0.625	0.541
Macro	0.558	0.580	0.559	0.604	0.616	0.604	0.678	0.695	0.683
Micro	0.608	0.576	0.582	0.652	0.626	0.633	0.750	0.727	0.734
Overall accuracy	0.576			0.626			0.727		
AUC (macro/micro)	0.772/0.778			0.818/0.799			0.864/0.879		
Delong test *p*-value	< 0.05			< 0.05			Reference

### Comparison of radiologists and the best radiomic model diagnosis capability

In the testing cohort of 139 patients, the performance of the AP&LAT model was better than that of the two radiologists, with accuracies of 0.727, 0.712, and 0.417 (Table 4), respectively. The final knee OA diagnostic radiomic model established in this study performed better than the radiologist with 2 years of musculoskeletal diagnostic experience (*p* < 0.05).

**Table 4:** Overall performance of radiologists and the best radiomic model based on testing cohort

	AP&LAT		Radiologist 1		Radiologist 2	
	Sensitivity	Specificity	Sensitivity	Specificity	Sensitivity	Specificity
Class 0	0.854	0.989	0.896	0.912	0.375	0.978
Class 1	0.676	0.924	0.765	0.829	0.176	0.752
Class 2	0.571	0.924	0.333	0.949	0.429	0.763
Class 3	0.75	0.924	0.700	0.933	0.500	0.974
Class 4	0.625	0.911	0.563	1.000	0.938	0.919
Overall accuracy (95% CI)	0.727 (0.645–0.799)		0.712 (0.629–0.786)		0.417 (0.334–0.504)	
*p*-value	Reference		0.084		< 0.05	

## Discussion

Radiographic imaging is frequently employed in the diagnosis of knee OA. Evaluating the severity of knee OA is a challenging and subjective process and often involves a qualitative analysis of plain radiographs. The K/L grading system, the most commonly utilized scale for classifying knee OA, is limited by its subjective nature and the notable variability in agreement between different observers. Therefore, an objective and consistent approach to grading the severity of knee OA is needed. Radiomics, the process of extracting quantitative features from medical images, holds promise for enhancing the precision and reliability of grading knee OA [17]. In our study, we devised a radiomic model utilizing the LR classifier to automate the grading of knee OA severity with plain radiographs. We selected both AP&LAT radiographs of the knee joint for the extraction of radiomic features. The mean ICC for the ROI delineation conducted by two radiologists was 0.91, signifying high reproducibility. The diagnostic efficiency results of all three radiomic models showed good diagnostic performance, and the AP&LAT model performed best, with the highest overall accuracy and the highest AUC value (macro/micro) of 0.864/0.879 in the test cohort, which was statistically significant compared to the other two models. The five-class ROC curves showed that the combined AP&LAT model achieved the best grading performance with AUCs of 0.990, 0.781, 0.728, 0.913, and 0.885 from class 0 to 4. These results suggest that combining information from both AP&LAT images significantly improved the performance of our model, and the combined AP&LAT radiomics model holds promise as a valuable tool for early and accurate diagnosis of knee OA.

Numerous studies have explored the application of radiomics in the classification and grading of knee OA. For instance, the research conducted by Abdelbasset Brahim et al [18] introduced a comprehensive computer-aided diagnosis (CAD) system designed for the early detection of knee OA utilizing knee X-ray images and machine learning algorithms. The findings revealed that the system offered promising predictive capabilities in OA detection, with an accuracy of 82.98%, a sensitivity of 87.15%, and a specificity reaching 80.65%. Likewise, the study by Mahrukh Saleem et al [19] showcased a computer-vision system aimed at aiding radiologists by assessing radiological indicators in X-rays for knee OA. The outcome demonstrated that this approach could effectively identify OA, achieving an impressive detection accuracy rate of over 97%. The above studies achieved good results in the identification of knee OA, but they all used the AP X-ray images and did not add the LAT radiography to the study. Additionally, a study by Luca Minciullo et al [20] introduced a fully automated technique utilizing a Random Forest Regression Voting Constrained Local Model (RFCLM) for differentiating between radiographs of individuals with knee OA and those without. The study highlighted that the automated analysis of the LAT view yielded classification results that were on par with or superior to those obtained by applying similar methods to the frontal view. This study showed that LAT images also have information for knee OA classification, although their study did not compare the classification effect of the AP, LAT, and combined images. In our prior research, we undertook a binary classification investigation of knee OA by developing a radiomic model. Our findings indicated that among the four groups of models tested, the LR model outperformed the others, achieving an AUC value of 0.843. This result demonstrates that the radiomics model possesses a strong capacity for the accurate diagnosis of knee OA [16]. Therefore, in this study, we continued to use the LR model to automatically grade knee OA and tested whether LAT radiographs could play an important role in model establishment. The results showed that the radiomics model could indeed accurately classify knee OA in radiographs, and the LAT images provided characteristic information that was different from the AP views.

The integration of deep-learning approaches, such as convolutional neural networks (CNNs), has enhanced the efficacy of radiomic models in the grading and classification of knee OA [21–23]. For instance, Berk Norman et al [24] introduced a fully automated algorithm called DenseNets, designed for knee OA detection employing K/L grading scales. The reported sensitivity rates for detecting no OA, mild, moderate, and severe OA were 83.7%, 70.2%, 68.9%, and 86.0%, respectively. Aleksei Tiulpin et al [25] conducted a study that yielded an automatic technique for predicting K/L and Osteoarthritis Research Society International (OARSI) grades from knee radiographs using deep learning. This method attained an impressive AUC of 0.98 and an average precision score of 0.98 in detecting the presence of radiographic OA. Kevin A. Thomas et al [26] created an automated model to assess the severity of knee OA using radiographic images. They compared the performance of their model with that of musculoskeletal radiologists. The model demonstrated an average F1 score of 0.70 and an accuracy of 0.71 across the entire test set. One of our previous studies also showed that deep-learning techniques can accurately grade knee OA in X-ray images and we also found that multiview X-ray images and prior knowledge improved classification efficacy. The overall accuracy of the DL model with multiview images and prior knowledge was 0.96 compared to 0.86 for an experienced radiologist [27]. Despite deep-learning techniques being regarded as cutting-edge technology for image classification, their highly complex internal structures often render the models’ decision-making processes opaque to human understanding, leading to a lack of explicability [28, 29]. However, the features extracted through radiomics are more interpretable, offering a clear understanding of their outcomes [10]. In our study, the feature extraction process captures a range of traceable image information that may elude radiologists’ observation but proves to be crucial for the diagnosis of knee OA.

Currently, the focus of international research on the musculoskeletal system is predominantly on osteoporosis, bone mineral density, fractures, bone tumors, and the like. The majority of investigations into degenerative osteoarthropathy are underpinned by CT/MRI imaging [25, 30]. Plain radiographs are seldom used in isolation due to their limited informational yield. But for knee OA, X-ray is a faster and more convenient non-invasive examination method. Faster and more accurate knee OA grading will help both radiologists and clinicians in their work. Abdelbasset Brahim et al confirmed that the radiomics model can accurately distinguish the K/L classification of the knee joint in X-ray images, with an accuracy of 82.98% [18]. This is consistent with our results, indicating that radiomics model is accurate for the K/L classification of the knee joint. In this study, we conducted a comprehensive analysis of the knee joint X-ray, capturing detailed information such as the femur, medial and lateral tibial condyles, patella, and corresponding joint space width. Through the selection of radiomics features, we identified the nine most relevant features, which may not be discernible to the naked eye but play a crucial role in measuring important parameters of knee OA.

It is noteworthy that our study not only utilized AP radiographs of the knee joint but also incorporated LAT radiographs. This approach sets our study apart from many other related studies, allowing for a more comprehensive analysis of the knee joint from different perspectives [18–20, 31]. LAT radiographs offer enhanced density and shape information, enabling a more comprehensive assessment of knee lesions. However, it is noteworthy that most knee OA studies have primarily utilized AP radiographs. This preference can be attributed to the fact that the reference standard K/L grading for knee OA is based on evaluations conducted in the AP position [31–33]. Despite this, recognizing the value of LAT radiographs, it is essential to explore their potential benefits and incorporate them into future research to further enhance the evaluation of knee OA. In order to enhance the extraction of radiomic features and capture more knee image information, we made an innovative addition of LAT radiographs to our study. Interestingly, we discovered that the utilization of the LAT view model alone yielded greater overall accuracy and a higher AUC value compared to the AP view model. This finding highlights the potential superiority of LAT radiographs in improving the diagnostic performance of knee imaging analysis. We suspect there are several potential reasons for the observed differences in the effectiveness of LAT and AP radiographs. Firstly, the patella, which is one of the bones most affected by knee OA, is more prominently visible in the LAT view compared to the AP view, where it can be obscured by the femur. This improved visibility in LAT radiographs allows for a better assessment of patellar involvement in the radiomics model. Secondly, LAT radiographs provide a better display of joint space and characteristic information related to osteophytes, which may differ from the information obtained from AP images. This additional information, unique to LAT radiographs, can contribute to more accurate grading judgments by the radiomics model. These factors suggest that incorporating LAT radiographs can offer valuable insights and complementary information to enhance the performance of radiomics models in assessing knee OA. Further research and validation may help elucidate the full potential and benefits of utilizing LAT radiographs in this context. Compared to the conventional practice of delineating the ROI using rectangular shapes, our approach involves segmentation along the entire knee edge. This innovative technique enables more precise extraction of radiomic features, resulting in improved filtration of irrelevant image information. Through the utilization of this segmentation technique, our aim is to enhance the discriminative power of the radiomics model, providing more reliable and meaningful insights into the assessment of knee OA. Nonetheless, further validation and comparative studies are warranted to comprehensively evaluate the benefits and potential advantages of this segmentation methodology over existing approaches.

Finally, we have demonstrated that the radiomic model developed in this study outperformed radiologists with 4 years and 2 years of musculoskeletal diagnostic experience. This finding indicates that our AP&LAT model exhibits higher accuracy compared to junior radiologists and can conveniently offer clinicians with diagnosis and treatment guidance.

Our study is subject to several limitations that should be acknowledged. Firstly, it is important to note that this study is based on a retrospective analysis, with all radiographic data obtained solely from a single hospital. This lack of diverse external data verification may introduce potential selective bias into our findings, thereby limiting the generalizability of our results. Secondly, the radiomics analyses in our study were conducted exclusively using radiographic images. Future studies would benefit from incorporating joint analyses of multimodal datasets and incorporating additional clinical parameters. This approach would provide a more comprehensive and holistic understanding of the disease and potentially improve the accuracy and robustness of the radiomic model. Thirdly, manual segmentation of the ROI for each image was performed, which can be time-consuming and may introduce inter-observer variability. Exploring the feasibility of automatic segmentation techniques in future research could significantly enhance efficiency and reduce potential errors in ROI delineation.

## Conclusion

Our study demonstrates that a radiomic model based on plain radiographs can accurately grade the severity of knee OA. The use of the LR classifier and the combination of information from both AP&LAT images significantly improves the performance of the model. This approach could potentially improve the accuracy and consistency of knee OA grading, which is important for clinical decision-making and treatment planning.

## Data Availability

The datasets are available from the corresponding author with a reasonable request.

## Abbreviations

AP: Anteroposterior
AUC: Area under the curve
ICC: Intraclass correlation coefficient
K/L: Kellgren–Lawrence
LAT: Lateral
LR: Logistic regression
OA: Osteoarthritis
ROC: Receiver operating characteristic
ROI: Region of interest
