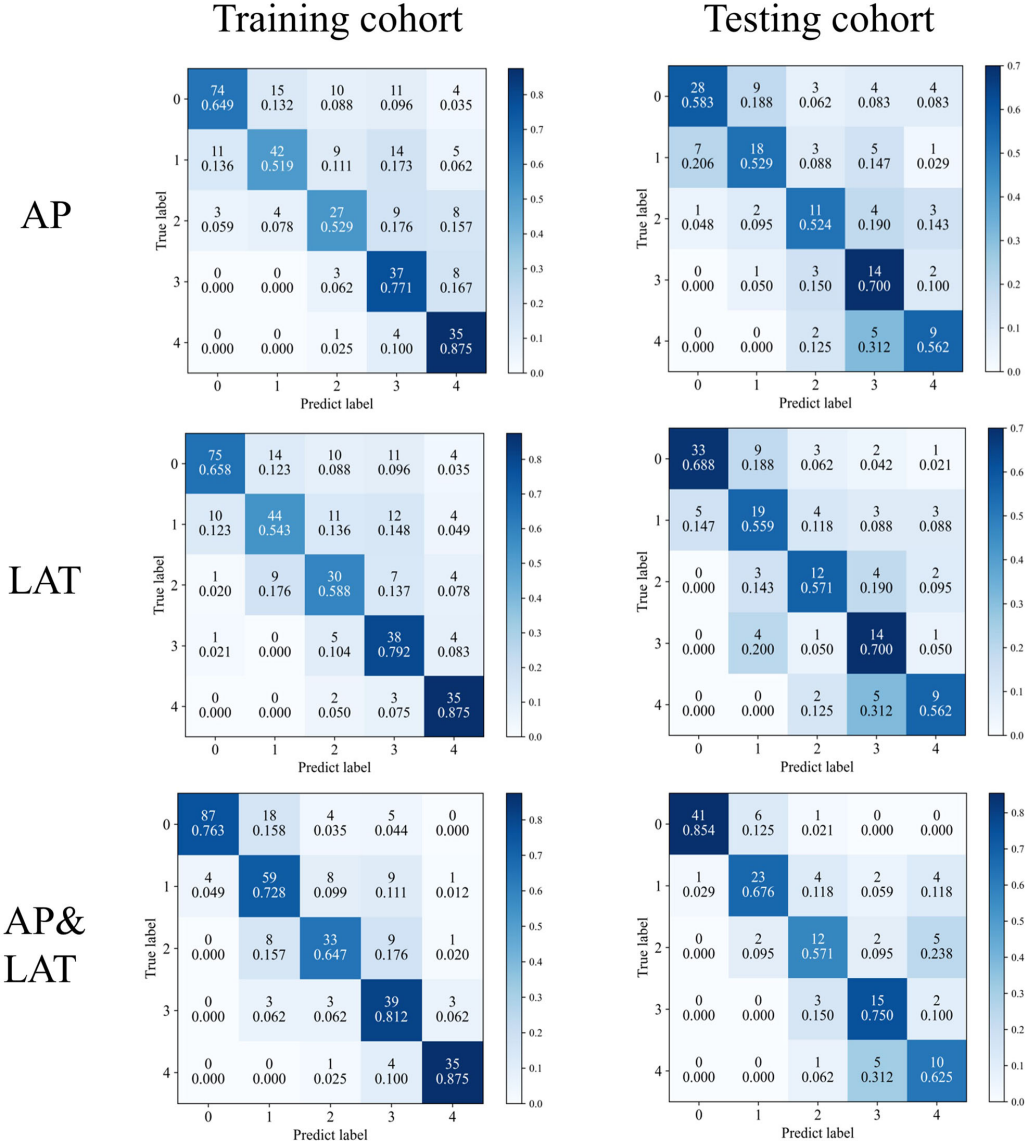# Correction: Automatic grading of knee osteoarthritis with a plain radiograph radiomics model: combining anteroposterior and lateral images

**DOI:** 10.1186/s13244-024-01773-x

**Published:** 2024-08-01

**Authors:** Wei Li, Jin Liu, Zhongli Xiao, Dantian Zhu, Jianwei Liao, Wenjun Yu, Jiaxin Feng, Baoxin Qian, Yijie Fang, Shaolin Li

**Affiliations:** 1 Department of Radiology, The Fifth Affiliated Hospital of Sun Yat-sen University, Zhuhai, Guangdong Province China; 2 Huiying Medical Technology (Beijing), Huiying Medical Technology Co., Ltd., Room A206, B2, Dongsheng Science and Technology Park, Haidian District, Beijing, 100192 China

**Correction to:** ***Insights into Imaging***

10.1186/s13244-024-01719-3, published online 13 June 2024

The original article erroneously displays Figure 5 without the LAT model in the test cohort.

The corrected version of Figure 5 with the LAT model present can be viewed ahead in this correction article.